# ALLOSTATIC LOAD AND STATUS IN MID LIFE AND BEYOND

**DOI:** 10.1093/geroni/igad104.2765

**Published:** 2023-12-21

**Authors:** Micah Bailey-Arafah

**Affiliations:** Case Western Reserve University, Cleveland, Ohio, United States

## Abstract

**Objective:**

This study explores how status and processes of cumulative dis/advantage shape allostatic load across the life course.

**Methods:**

Data from these analyses are from a national sample of adults from the Biomarker Substudy of the Study of Midlife in the US (MIDUS), a longitudinal study of health and well-being in the United States (N=1186) aged 35-86 years old. Education level, multiple indicators of financial stability, and demographic data were used to construct a measure of status. Those in the Biomarker substudy completed a physiological assessment that included blood, urine, and saliva assays to assess functioning of the sympathetic and parasympathetic nervous systems, hypothalamic-pituitary-adrenal axis, cardiovascular, inflammatory immune activity, glucose metabolic system, and lipid metabolic system. These data were used to create an allostatic load index. An alternate allostatic load index was developed using reported medication to affect three of seven biological systems: cardiovascular and the glucose and lipid metabolic systems. Nested regression models were estimated to explain differences in allostatic load.

**Results:**

Reported financial instability across the life course was associated with higher levels of allostatic load, and higher levels of education were associated with lower levels of allostatic load, both with and without reported medication usage.

**Conclusion:**

These findings demonstrate that structural forces impact allostatic load, both with and without reported medication usage, and expand previous work on allostatic load and social status.

